# How to Enhance Adherence in Patients with Nonalcoholic Fatty Liver Disease: A Thought-Provoking Issue

**DOI:** 10.2196/59208

**Published:** 2024-05-14

**Authors:** Chunlan Liu, Run Zhou, Junping Shi

**Affiliations:** 1 School of Nursing Hangzhou Normal University Hangzhou China; 2 Department of Infectious Disease and Hepatology The Affiliated Hospital of Hangzhou Normal University Hangzhou China; 3 Zhejiang Key Laboratory of Medical Epigenetics Hangzhou China; 4 Institute of Hepatology and Metabolic Disease Hangzhou Normal University Hangzhou China

**Keywords:** NAFLD, adherence, digital therapeutics, lifestyle modification, mobile health, nonalcoholic fatty liver disease, self-management, randomized controlled trial

We read with great interest the article by Kwon et al [1] in the *Journal of Medical Internet Research* that described an app for lifestyle coaching intervention for patients with nonalcoholic fatty liver disease (NAFLD). With the paradigm shift in medicine, we are delighted to find that the study took into account the importance of psychological and social factors for patients. Digital therapeutics have shown remarkable contributions in various fields [2]. Our team has done some research on the use of digital therapeutics in NAFLD [3]. We consider that the remote intervention developed by researchers based on the ADDIE (Analysis, Design, Development, Implementation, and Evaluation) model can be regarded as a form of digital therapeutics. Having closely examined the research, we were inspired by several findings.

In this research, patients with adherence of 90% or more had improved BMI, liver fat score, and alanine transferase after 6 months of intervention compared to patients with adherence of less than 90%. In addition, participants in the higher adherence group also showed the highest performance in the areas of self-management and knowledge [1]. This shows that adherence plays a crucial role in the out-of-hospital treatment and management of NAFLD.

However, in terms of assessment, the researchers only evaluated participant adherence to the intervention through dietary recording rates (82.6% at 3 months and 79.8% at 6 months) [1]. We were concerned about whether this could give rise to a one-sided assessment. According to our knowledge, adherence assessments for mHealth interventions typically encompass a broader range of metrics, including completion of multifunctional modules, frequency of application visits, and the number of self-reported activities [4]. Therefore, in order to accurately assess patient adherence, researchers should update and refine subsequent iterations of the SMART-Liver app.

Furthermore, it is worth noting that while the average adherence was good, only 15 patients maintained adherence levels above 90% [1]. In addition, our team recently conducted a behavioral survey among 380 adults with NAFLD and found significant room for improvement in treatment adherence for the majority of patients [5]. With that in mind, here are some suggestions. On the one hand, researchers could personalize the management program by matching participants’ characteristics such as age, gender, education, dietary preferences, and movement disorders before the intervention. This approach would tailor the program to individual needs and potentially enhance adherence. On the other hand, fostering interest is essential for long-term adherence. Therefore, we suggest that the app be designed with attractive functional sections, such as artificial intelligence exercise coaches, virtual therapists, and interactive knowledge games. These features could engage users and motivate them to adhere to their treatment regimen more consistently [3].

This research represents a breakthrough in the out-of-hospital treatment and management of NAFLD. Having used the features of digital therapeutics to their fullest potential, researchers have to not only be free from the constraints of time and space but also bring substantial economic advantages to stakeholders. Undoubtedly, this development holds immense significance for NAFLD patients, health care professionals, and research and development companies.

## Abbreviations

ADDIE: Analysis, Design, Development, Implementation, Evaluation
NAFLD: nonalcoholic fatty liver disease
